# The Gastrointestinal Irritation of Polygala Saponins and Its Potential Mechanism In Vitro and In Vivo

**DOI:** 10.1155/2015/918048

**Published:** 2015-02-01

**Authors:** Li Wen, Nan Xia, PeiPei Tang, Yi Hong, ZiZhen Wang, YaJie Liu, YanJu Liu, JianJun Liu, XiangQiong Li

**Affiliations:** ^1^Ministry of Education Key Laboratory of Traditional Chinese Medicine Resources and Compounds, Hubei College of Traditional Chinese Medicine, Wuhan, Hubei 430061, China; ^2^Wuhan Institute of Biological Products Co., Ltd., Wuhan, Hubei 430207, China

## Abstract

Processing alters the pharmacological activity and reduces the gastrointestinal toxicity of the polygalae. To investigate the effect of processing, different glycosyl substituent products were tested. Hypnotic and subhypnotic doses of pentobarbital-induced sleep tests on mice were used to evaluate the sedative activity of polygala saponins with different glycosyl substituents; isolated gut motility experiment was employed to study excitatory effects of different polygala saponins; the gastrointestinal irritation effects of different polygala saponins were compared by measuring the levels of gastric PGE2 and intestinal TNF-*α* on mice. When compared with control, Onjisaponin B (OJB) and tenuifolin (TEN), but not senegenin (SNG), significantly increased the number of sleeping mice and prolonged the sleeping time (*P* < 0.05); 80, 40, and 20 mg/L of OJB and 80 mg/L of TEN, but not SNG, obviously changed the amplitude and frequency of isolated jejunum (*P* < 0.05); all the three compounds significantly decreased the level of gastric PGE2 but had no obvious influences on the reduction of intestinal TNF-*α* level. For sedative and hypnotic effects, OJB > TEN > SNG; for the protection form gastrointestinal irritation and damages, OJB > TEN > SNG. Therefore, in processing Polygala, glycosyl breaking may be related to the decline of pharmacological activity and gastrointestinal toxicity of polygala saponins.

## 1. Introduction

**Figure 1:**
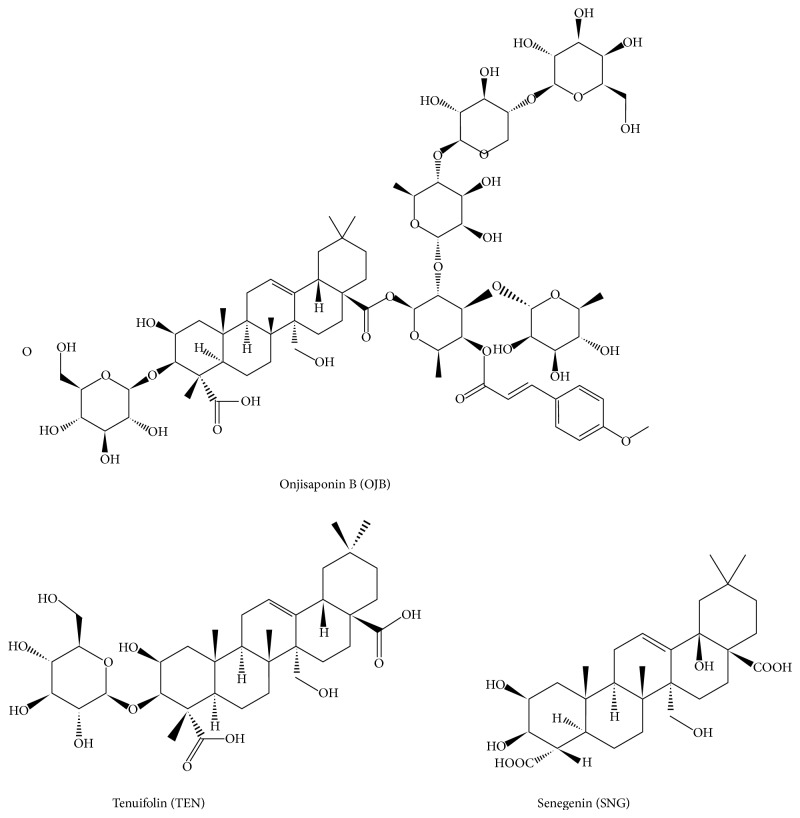
Molecular structure of Onjisaponin B (OJB).

Radix Polygalae comes from the dried roots of* Polygala tenuifolia* willd. or* Polygala sibirica* L. [1], which is often used clinically for insomnia, forgetfulness, phlegm, palpitation, and so forth [2]. However, research shows that long-term and large doses of Radix Polygalae can cause gastric mucosal injury [3–5]. Nonprocessed Radix Polygalae caused obvious inhibition of gastrointestinal motility and led to flatulence, thinner, or necrosis intestinal [6–8]. Pharmacy workers always process Radix Polygalae to reduce gastrointestinal irritation, such as honey-stir-baked [9, 10]. The major component of total saponins in Radix Polygalae is triterpenoid saponin [11–18], whose nuclear parent is senegenin (SNG). In the process of extraction, the glycosyl link at C28 breaks easily and then produces more TEN [5], and the glycosyl link at C3 breaks and becomes SNG (Figure 1).

So, we selected three compounds, namely, Onjisaponin B (OJB), TEN, and SNG to evaluate the sedative activity of the three compounds using hypnotic and subhypnotic doses of pentobarbital-induced sleep test on mice, to study excitatory effects of them by employing isolated gut motility experiment, and to compare the toxic effects of them by measuring the levels of gastric PGE2 and intestinal TNF-*α* in mice.

## 2. Materials and Methods

### 2.1. Materials

HPLC with UVD170U detector, quaternary low pressure gradient pump (Dionex-P680), purchased from Dionex Company (USA).

Radix Polygalae was purchased from Wuhan Medicinal Materials Company (Wuhan, China) and identified by Dr. Wenjie Tang (Department of Pharmacology, Hubei College of Traditional Chinese Medicine, Wuhan, China). The voucher specimen was deposited at the Herbarium of Hubei college of Traditional Chinese Medicine under the acquisition number of WWZ0406.

SNG and TEN (>98% purity) were purchased from Chengdu Pusi Co., Ltd. (Chengdu, China). OJB was isolated and purified by Ministry of Education Key Laboratory of Traditional Chinese Medicine Resources and Compounds, Hubei College of Traditional Chinese Medicine.

Pentobarbital sodium (Lot number: F20030816) was provided by Sinopharm Shanghai Chemical Reagents Co., Ltd. (Shanghai, China); estazolam (Lot number: 20111029) was from Anhui Bangning Pharmaceuticals (Anhui, China). ELISA assay kits for PGE2 and TNF-*α* were purchased from Sigma Chemicals Co. (St. Louis, MO, USA). Acetylcholine (Lot number: 091201) was purchased from Shanghai Institute of Biochemistry, CAS (Shanghai, China). BaCl2 (Lot number: 20110221) was purchased from Tianjin Deen Chemical Reagent Co., Ltd. (Anhui, China). Phentolamine (Lot number: 101104) was purchased from Shanghai Fudan Forward Science and Technology CO., Ltd. (Shanghai, China). Atropine (Lot number: 1005051) was purchased from Zhengzhou Lingrui Pharmaceutical Co., Ltd. (Zhengzhou, China). Neostigmine (BCBD8573V) was purchased from Sigma-Aldrich Co. (St. Louis, MO, USA).

### 2.2. Preparation of Test Samples

**Figure 2:**
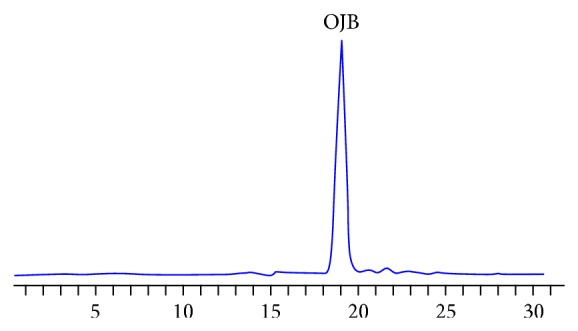
Measurement of Onjisaponin B (OJB) content by HPLC, the content of OJB is 95%.

Preparation of PTS [1]: 1 kg of pieces of Polygala tenuifolia. Wild were extracted three times (2 h/time) by 3-fold volumes of 85% EtOH, and the extracted solution was pooled. Ethanol was recycled by evaporating until there was no alcohol smell and then was dissolved in 1000 mL of water. Acetic ether saturated by water was extracted 3 times (300 mL/time) and acetic ether was discarded and combined with aqueous phase. Water was added to 1000 mL then eluted on a D101 macroporous resin column with H_2_O, 30% EtOH and 70% EtOH, using 3 times the column volume, 1 BV/h. The fraction eluted by 70% EtOH was condensed until there was no alcohol smell, to obtain powder A (the yield was 5.32 g/100 g of dried medicinal herbs). The content of total saponins was identified to reach to 52% (calculate by the content of tenuifolin (TEN)) [13]; powder A was eluted by HPLC with preparative column, and the specific parameters were as follows [19–22]: the mobile phase of water containing 0.5% acetic acid and acetonitrile (38 : 62), C18 column (250∗4.6 mm), and evaporative light-scattering detector. After the above steps, compound B was obtained and measured by HPLC (C18 column (250∗4.6 mm) area normalization method was above 95%, the mobile phase of methanol and water containing 0.05% phosphoric acid solution (65 : 35)). As shown in Figures 1 and 2, compound B was further identified to be polygala saponins B (OJB, C75H112O35) by ^13^C and ^1^H NMR as well as infrared spectrometry (Figure 1) [23].

Estazolam (EST) was dissolved by normal saline to prepare the solution with concentration of 0.5%.

Preparation of OJB, TEN, and SNG solution was as follows: for investigating pharmacological effects on mice, all three compounds were dissolved by normal saline (containing 0.5% Tween 80) to prepare the solution with a concentration of 20 mg/mL, respectively; the high concentration diluted to 20 mg powdered material per mL as the low concentration for mice; the dosing volume for mice was 10 mL/kg. For determining gastrointestinal irritation powdered OJB, TEN, or SNG dissolved in water (containing 0.5% Tween 80) and diluted to a concentration of 20 mg powdered material per mL as the high concentration for mice. The solution was prepared and diluted to different concentrations (8, 4, and 2 mg/mL) for isolated gut motility experiment; 200 uL was added into per 20 mL Tyrode's solution.

Preparation of polygala total saponins (PTS) was as follows: powdered PTS dissolved in water (containing 0.5% Tween 80) and diluted to a concentration of 20 mg powdered material per mL as the high concentration for mice; the solution was prepared and diluted to different concentrations (8, 4, and 2 mg/mL) for isolated gut motility experiment; 200 uL was added into per 20 mL Tyrode's solution.

### 2.3. Animals

Male Kunming mice weighing 18–22 g and male rabbit weighing 2–2.5 kg were procured from the Center for Disease Prevention and Control in Hubei Province, China (reg. number SCXK (Hubei) 2008-0005). The animals were housed at 22 ± 2°C under a 12 h light/12 h dark cycle and with access to food and water ad libitum. The mice were acclimatized and habituated to the laboratory for at least a week before being subjected to tests and were used only once throughout the experiments. The study was carried out along with the “Principles of Laboratory Animal Care” [24]. The experiment was performed according to the guidelines of the Committee for the Purpose of Control and Supervision of Experiments on Animals and approved by the Animal Experiment Ethical Committee of Hubei University of Chinese medicine.

### 2.4. Potentiation of SPB, TEN and SNG on Pentobarbital-Induced Sleep in Mice

Male Kunming mice were randomly divided into 8 groups (8 mice per group). In the control group, mice received equal volume of normal saline, while in the seven other groups, mice were intragastrically treated with 200 mg/kg of OJB, 400 mg/kg of OJB, 200 of mg/kg TEN, 400 of mg/kg TEN, 200 of mg/kg SNG, 400 mg/kg of SNG, and 400 mg/kg of PTS for one time 30 min before pentobarbital treatment. 40 mg/kg pentobarbital was used as hypnotic dose to treat half of the animals, and the others were administered with 25 mg/kg which was used as subhypnotic dose [25–29]. The subhypnotic dosed mice were observed for sleep onset within 15 min; mouse losing righting reflex over 1 min was considered to be asleep. The sleeping time of animals receiving hypnotic dose was also observed. The sleeping latency was recorded from the injection of pentobarbital to the sleep onset and the sleeping time was recorded from the loss of righting reflex to recovery. The results were shown in Tables 1 and 2.

### 2.5. Influence of Different Polygala Saponins on Isolated Gut Motility of Rabbit

The rabbit ileum was dissected out and cut into 2 cm segment for preparing isolated intestinal smooth muscle sample according to previous description [30]. The normal motion curve of intestinal segment was recorded when spontaneous rhythmic contraction reached to equilibrium. Different solutions with a volume of 0.2 mL were added into the bath in order: acetylcholine (1 : 10000), BaCl_2_ (20%), dopamine (10 mg/mL), phentolamine (10 mg/mL), atropine (0.5 mg/mL), and neostigmine (10 mg/mL). The segments with normal contraction after drug stimulation were subjected to the following experiment. The changes of curve before and after compound treatment were recorded. Specifically, the mean value of tension within 3 min interval before and after treatment was obtained to calculate the changing ratio as follows.

 Increasing of intestinal tension (IIT) = Mean of tension_after  treatment_ − Mean  of  tension_before  treatment_.
 Changing ratio of intestinal tension (CRIT) = (Mean of tension after treatment − Mean of tension before treatment)/Mean of tension before treatment × 100%.

**Figure 3:**
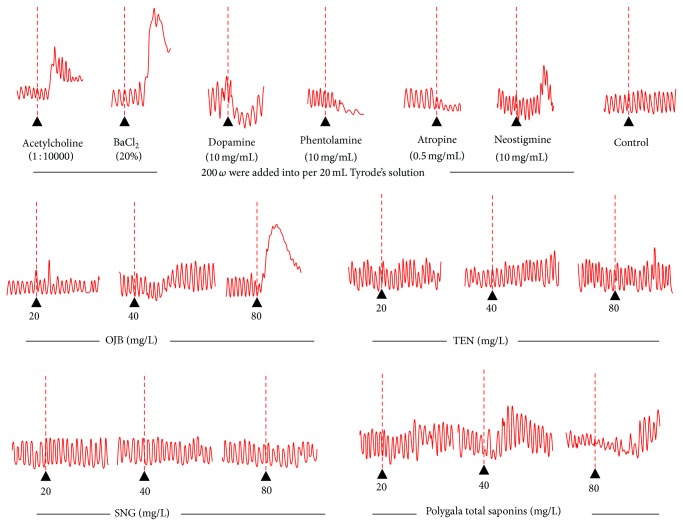
Effects of Onjisaponin B (OJB), tenuifolin (TEN), and senegenin (SNG) on isolated gut motility in rabbit. Mark: the solution was prepared and diluted to different concentrations (8, 4, and 2 mg/mL) for isolated gut motility experiment, 200 uL were added into per 20 mL Tyrode's solution, measuring the average tension 3 min before and after the administration by the method of interval measurement.

The results were shown in Table 3 and Figure 3.

### 2.6. Influence of Different Polygala Saponins on Gastric PGE2 and Intestinal TNF-*α* Levels

**Figure 4:**
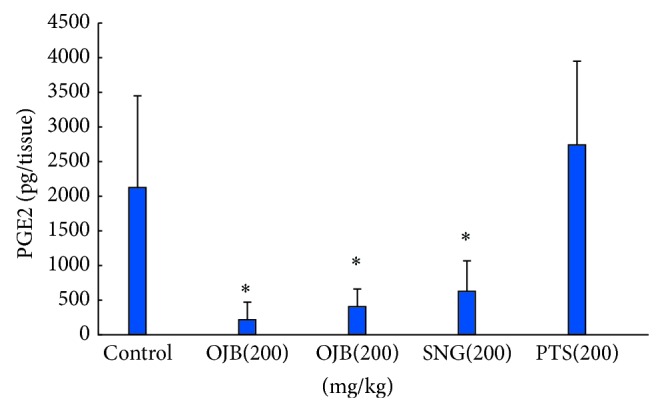
Influence of polygala saponins with different glycosyl on gastric PGE2 levels in mice. Mark: mice were, respectively, administrated by intragastrical administration (I.G) with the doses (200 mg/kg) of Onjisaponin B (OJB), tenuifolin (TEN), and senegenin (SNG); negative control groups were treated with equal volume of vehicle. After the gastrointestinal tissues in mice were homogenized, we determine PGE2 concentration of gastric homogenate. Each column is mean ± standard deviation for 10 mice in each group. ^*^ *P* < 0.05 versus control.

**Figure 5:**
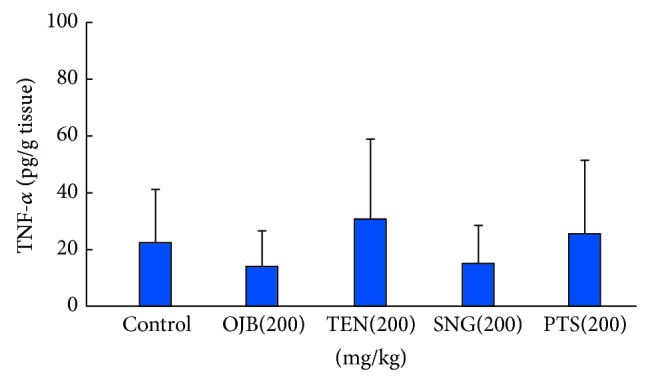
Influence of polygala saponins with different glycosyls on gastric TNF-*α* levels in mice. Mark: mice were, respectively, administrated by intragastrical administration (I.G) with the doses (200 mg/kg) of Onjisaponin B (OJB), tenuifolin (TEN), and senegenin (SNG). Negative control groups were treated with equal volume of vehicle. After the gastrointestinal tissues in mice were homogenized, we determine TNF-*α* levels of gastric homogenate. Each column is mean ± standard deviation for 10 mice in each group.

Male Kunming mice were randomly divided into 5 groups (10 mice per group): control, OJB, TEN, SNG, and polygala total saponins. Animals were intragastrically treated with 200 mg/kg of OJB, TEN, SNG, and total polygala saponins for one time, while the control mice received equal volume of normal saline (except polygala total saponins group, animals are the ones given 25 mg/kg pentobarbital in 2.4). After 30 min, the animals were sacrificed and the stomachs and intestines were dissected out and stored at −80°C. 1 mg of tissues were homogenized in ice-cold buffer and then subjected to the ELISA measurement of gastric PGE-2 and intestinal TNF-*α* levels. The results were shown in Figures 4 and 5.

### 2.7. Statistical Analysis

All data are presented as means ± S.E.M. One-way ANOVA was first used to assess the differences among multiple groups, followed by the Dunnett post hoc test. The significance of normal and treated groups was using the student's *t*-test. Only for subhypnotic dosage of pentobarbital-treated test, chi- square test was used to compare the number of falling asleep between control group and each of other groups. The data was assessed by the statistical package from the Social Science Software (SPSS) program. The levels of significance are indicated by different letters.

## 3. Results

### 3.1. Effects of Different Polygala Saponins on Pentobarbital-Induced Sleep in Mice

As shown in Table 1, both OJB and TEN, but not SNG, significantly prolonged pentobarbital-induced sleeping duration with hypnotic dose when compared with control (*P* < 0.05). Therefore, OJB and TEN could potentiate pentobarbital-induced sleep by prolonging sleeping duration.

As shown in Table 2, both OJB and TEN significantly increased the number of sleeping mice when compared with control (*P* < 0.01), indicating a potentiated effect of OJB and TEN on pentobarbital-induced sleep. However, SNG had no obvious effect on the number of sleeping mice when compared with control (*P* > 0.05).

### 3.2. Influence of Different Polygala Saponins on Isolated Gut Motility of Rabbit

As shown in Table 3, when compared with control, 80, 40, and 20 mg/L of OJB increased the intestinal tension by 48.13%, 20.49%, and 12.84% within 30 s, respectively (*P* < 0.05, *P* < 0.01, or *P* < 0.001). 80 mg/L of OJB elevated the intestinal tension by 1.04 g within 30 s comparing to the control (*P* < 0.05). The contractive amplitude presented irregular results, as shown in Figure 3; SNG exhibited no significant influences on tension, amplitude, and frequency. Therefore, only OJB had the most profound impact on gastrointestinal motility.

### 3.3. Influence on Gastrointestinal PGE2 and TNF-*α* Levels

As shown in Figure 4, when compared to control, 200 mg/kg of OJB, TEN, or SNG could significantly reduce the gastric PGE2 level (*P* < 0.05), and the effects of OJB were greater than that of TEN (*P* < 0.05).

As shown in Figure 5, when compared to control, 200 mg/kg of OJB, TEN, and SNG had no significant effect on TNF-*α* level (*P* > 0.05).

## 4. Discussion

In routine clinical usage, Radix Polygalae can cause mild nausea. If it is taken in larger doses, it can cause nausea, vomiting, abdominal distension, diarrhea, and adverse reaction of hemolysis. In China, pharmacy workers always process Radix Polygalae to reduce gastrointestinal irritation, such as honey-stir-baked [9], because in the course of processing drugs, the glycosyl link at C28 of PTS breaks easily and then produces more tenuifolin (TEN), that is, glycosyl at C3, which becomes senegenin (SNG) on hydrolysis under alkaline condition (Figure 1). We selected these three compounds to identify the effects of glycosyls on pharmacological and toxic effects of polygala saponins. We take the following experiments: (a) hypnotic and subhypnotic doses of pentobarbital-induced sleep tests were used to evaluate the sedative activities of these three compounds; (b) isolated gut motility experiment was employed to compare the gastrointestinal irritation of these three compounds; and (c) the toxic effects of different polygala saponins were determined by measuring the levels of gastrointestinal mucosa protecting factor PGE2 and inflammatory cytokine TNF-*α*.

This study compared the activities of them in the pentobarbital-induced sleeping experiment. The results showed both OJB and TEN potentiated pentobarbital-induced sleeping effect and also prolonged the sleeping duration, but the activities of SNG were limited. It is suggested that the presence of glycosyl may be critical for the activity of saponins. This phenomenon is very common in traditional medicine. For example, rhubarb dianthrone glycosides, the active component of rheum, is hydrolyzed to aglycone in the large intestine then leading to purgation while the aglycone in rheum has no such purgative effect, and the major reason is due to the stereospecific blockade of glycosyls in dianthrone glycosides which protects it from gastric acid destruction. Therefore, it could be deduced that the glycosyls in polygala saponins may have the function that protects it from being destroyed by gastric acid.

The results of isolated gut motility test revealed that OJB had much more profound impact on gastrointestinal tension than TEN, whereas SNG had almost no effect on isolated gut, and OJB of 80 mg/L can cause irregular and intense systole of isolated gut. All the above indicate that the amount of glycosyl might highly correlate with increased gastrointestinal irritation [31].

PGE2 is a protector of gastric mucosa. However, it is also an inflammatory factor. OJB and TEN could significantly reduce the level of gastric PGE2, which results in the deprivation of protection to gastric mucosa and finally causes gastric injury. SNG, without glycosyl, has a weaker effect on the reduction of gastric PGE2 level than OJB and TEN dose. We speculate that the presence of glycosyl could irritate gastrointestinal itself, which may be associated with the gastric mucosa injury caused by saponins.

TNF-*α* accelerates chemotaxis and necrosis of macrophage and also promotes inflammation. In the present study, OJB, TEN, and SNG could not significantly reduce the TNF-*α* level, suggesting that polygala saponins with glycosyls caused gastrointestinal irritation via reducing PGE2 level as well as its gastrointestinal protection but not generating inflammatory mediator (TNF-*α*).

Combined with many researchers' study, we infer that polygala saponins cannot cause gastrointestinal ulcer by producing inflammatory irritation. The use of a high dose of Polygala saponins leads to gastrointestinal congestion, swelling, which is mainly produced by decreasing the gastric mucosal protective factor PGE2 and has the characteristic of nonsteroidal anti-inflammatory drugs. Thus, we infer that it may have anti-inflammatory effect, making it possible to be against Alzheimer's, hypertension, and so forth. The strength of three compound's Sedative activities is OJB > TEN > SNG, and the strength of gastric ulcers irritation caused by reducing the content of gastrointestinal PGE2 is also OJB > TEN > SNG. In summary, just as our anticipation, when Radix Polygalae was fried with honey, it should have something to do with the fact that Radix Polygalae saponin hydrolyzes to TEN and SNG. So Radix Polygalae saponin's irritation to gastric mucosa decreased, and its sedative effects decreased, too. The present study also demonstrated TEN had favorable sedative and hypnotic activities and possessed lower gastrointestinal irritation than OJB, which made TEN to be a potential substitute for polygala.

## Figures and Tables

**Table 1:** Effects of polygala saponins with different glycosyl on pentobarbital-induced sleeping duration in mice.

Groups	Dose (mg/kg)	Percentage of falling asleep (%)
Control	—	10
OJB	200	70^a^
	100	70^a^
TEN	200	60^a^
	100	70^a^
SNG	200	30
	100	20
EST	1	80^2a^

Mark: mice were respectively administrated by intragastrical administration (I.G) with high and low doses (200 and 100 mg/kg) of the Onjisaponin B (OJB), tenuifolin (TEN), senegenin (SNG), and 1 mg/kg of estazolam (EST) for one time 30 min before pentobarbital treatment. Mice were administered with 25 mg/kg which was used as subhypnotic dose. Negative control groups were treated with equal volume of vehicle. *n* = 10. ^a^ *P* < 0.05; ^2a^ *P* < 0.01 versus control.

**Table 2:** Effects of polygala saponins with different glycosyls on pentobarbital-induced sleeping animal amount in mice.

Groups	Dose (mg/kg)	Sleep latency	Sleeping time (min)
Control	—	8.2 ± 2.0	18.2 ± 9.0
OJB	200	7.8 ± 2.6	35.6 ± 11.3^a^
	100	7.0 ± 1.7	39.7 ± 14.2^a^
TEN	200	8.4 ± 3.4	31.2 ± 7.4^a^
	100	9.0 ± 2.9	28.6 ± 3.5^a^
SNG	200	8.7 ± 2.9	27.1 ± 11.1^a^
	100	7.9 ± 2.6	18.8 ± 11.0
EST	1	9.4 ± 4.1	26.8 ± 4.6^a^

Mark: mice were, respectively, administrated by intragastrical administration (I.G) with high and low doses (200 and 100 mg/kg) of the Onjisaponin B (OJB), tenuifolin (TEN), senegenin (SNG), and 1 mg/kg of estazolam (EST) for one time 30 min before pentobarbital treatment. Mice were administered with 40 mg/kg. Negative control groups were treated with equal volume of vehicle. *n* = 10. ^a^ *P* < 0.05 versus control.

**Table 3:** Influence of polygala saponins with different glycosyls: Onjisaponin B (OJB), tenuifolin (TEN), and senegenin (SNG) on isolated intestinal tension in rabbit in vitro.

Groups	Concentration (mg/L)	IIT (g)	CRIT (%)	Amplitude (g)
Control	—	0.04 ± 0.29	−0.53 ± 8.21	1.96 ± 0.55

SNG	20	−0.12 ± 0.26	−0.14 ± 12.98	1.52 ± 0.31
	40	−0.02 ± 0.37	1.60 ± 13.75	2.11 ± 0.43
	80	0.27 ± 0.49	8.31 ± 16.14	2.02 ± 0.93

TEN	20	0.09 ± 0.80	−3.06 ± 30.22	2.03 ± 0.84
	40	0.15 ± 0.30	−3.74 ± 11.36	1.87 ± 0.50
	80	0.57 ± 0.53^a^	18.28 ± 26.44	2.11 ± 0.44

OJB	20	0.25 ± 0.42	12.84 ± 15.34^a^	2.38 ± 0.56
	40	0.49 ± 0.28^2a^	20.49 ± 16.61^2a^	2.25 ± 0.31
	80	1.04 ± 0.62^3a^	48.13 ± 34.39^3a^	2.36 ± 0.71

PTS	20	−0.03 ± 0.52	−2.18 ± 18.07	1.82 ± 0.27
	40	0.26 ± 0.51	8.96 ± 16.54	2.33 ± 0.54
	80	0.58 ± 0.47^2a^	19.2 ± 8.47^3a^	1.90 ± 0.43

Mark: the solution was prepared and diluted to different concentrations (8, 4, and 2 mg/mL) for isolated gut motility experiment; 200 uL was added into per 20 mL Tyrode's solution, measuring the average tension 3 min before and after the administration by the method of interval measurement. (*n* = 8, mean ± S.E.M.). ^a^ *P* < 0.05; ^2a^ *P* < 0.01; ^3a^ *P* < 0.01 versus control. Increasing of intestinal tension (IIT) = Mean of tension_after treatment_  −  Mean of tension_before treatment_;

Changing ratio of intestinal tension (CRIT) = (Mean of tension_after treatment_  −  Mean of tension_before treatment_)/Mean of tension_after treatment_  ×  100%.
